# Inferring Gene Function and Network Organization in *Drosophila* Signaling by Combined Analysis of Pleiotropy and Epistasis

**DOI:** 10.1534/g3.113.005710

**Published:** 2013-05-01

**Authors:** Gregory W. Carter

**Affiliations:** The Jackson Laboratory, Bar Harbor, Maine 04609

**Keywords:** genetic interaction, pleiotropy, epistasis, genetic network, signaling network

## Abstract

High-throughput genetic interaction screens have enabled functional genomics on a network scale. Groups of cofunctional genes commonly exhibit similar interaction patterns across a large network, leading to novel functional inferences for a minority of previously uncharacterized genes within a group. However, such analyses are often unsuited to cases with a few relevant gene variants or sparse annotation. Here we describe an alternative analysis of cell growth signaling using a computational strategy that integrates patterns of pleiotropy and epistasis to infer how gene knockdowns enhance or suppress the effects of other knockdowns. We analyzed the interaction network for RNAi knockdowns of a set of 93 incompletely annotated genes in a *Drosophila melanogaster* model of cellular signaling. We inferred novel functional relationships between genes by modeling genetic interactions in terms of knockdown-to-knockdown influences. The method simultaneously analyzes the effects of partially pleiotropic genes on multiple quantitative phenotypes to infer a consistent model of each genetic interaction. From these models we proposed novel candidate Ras inhibitors and their Ras signaling interaction partners, and each of these hypotheses can be inferred independent of network-wide patterns. At the same time, the network-scale interaction patterns consistently mapped pathway organization. The analysis therefore assigns functional relevance to individual genetic interactions while also revealing global genetic architecture.

The systematic study of genetic interactions has proven a powerful means to map genetic networks (Boone *et al.* 2007). Genome-scale interaction analysis has provided a global view of gene function in yeast (Costanzo *et al.* 2010), and studies focused on specific processes have mapped large-scale networks in yeast (Collins *et al.* 2007; Drees *et al.* 2005; Segre *et al.* 2005; St Onge *et al.* 2007), worm (Byrne *et al.* 2007; Lehner *et al.* 2006), and fly (Horn *et al.* 2011; Yamamoto *et al.* 2009). Analyses of statistical epistasis, the population-level manifestation of genetic interaction, have identified important effects in mouse (Li and Churchill 2010; Reifsnyder *et al.* 2000; Shao *et al.* 2008) and human (McKinney and Pajewski 2011; Ritchie 2011) genetics. These studies indicate that genetic interactions reveal underlying structure in biological networks and map complex genetic architecture. Advances in study design and the characterization of genetic populations have been accompanied by parallel progress in quantitative phenotyping. Multidimensional phenotypic characterization is becoming increasingly common, often including multiple physiological traits coupled with thousands of molecular measures such as protein and transcript abundances (Andreux *et al.* 2012; Chen *et al.* 2012). Such research ultimately aims to provide a genetically precise and phenotypically predictive approach to medicine. Success of this approach is contingent on the development of analytical methods to extract quantitative models from genetic interactions across multiple phenotypes. These methods will increase the power to formulate precise biological hypotheses to potentially address the complex genetics that underlie human health and disease.

To date, studies have primarily used statistical concordance of interaction patterns across multiple genes to infer the role of previously uncharacterized genes. This strategy is often referred to as guilt-by-association (GBA). Advanced GBA approaches, such as clustering genes based on correlated interaction spectra across multiple interaction partners (Carter *et al.* 2009; Collins *et al.* 2007; Costanzo *et al.* 2010; Drees *et al.* 2005; Segre *et al.* 2005), have successfully mapped genetic architecture on a large scale. In these networks genes often form highly connected communities, or gene modules, that are enriched in one or more functional annotations. The principle of GBA dictates that a minority of uncharacterized genes within a module can be assigned the dominant function of the module.

While successful on a large scale, GBA-based methods have multiple limitations. First, they require large data sets to generate adequate statistical power to resolve modules, and can therefore be limited in populations with a small number of relevant mutations such as studies of specific developmental or signaling processes, drivers of cancer evolution, or interacting candidates in genome-wide associations. Second, GBA relies on the availability of functional annotations for the vast majority of interacting genes. Third, GBA approaches often generate implicit predictions of gene function without providing explicit predictions of the effects of a mutation or combination of mutations, thereby limiting the power to generate directly testable hypotheses. Fourth, large-scale GBA approaches rarely take advantage of the complementary information in multiple phenotypes. In cases when multiple phenotypes are considered, the analysis is usually based on coincidence of interactions derived independently for each phenotype (Horn *et al.* 2011; Michaut and Bader 2012). Finally, it has been proposed that GBA results may be driven by a small number of critical interactions and therefore network associations are not generally reliable (Gillis and Pavlidis 2012).

Here we use an approach based on the combined analysis of pleiotropy and epistasis to infer the genetic architecture of growth-related signaling in *Drosophila melanogaster*. This strategy infers and interprets genetic interaction data in terms of quantitative variant-to-variant and variant-to-phenotype influences, rather than non-directional epistasis. This results in a network model that maps how each specific variant affects each other and, in turn, multiple related phenotypes. This method is applicable to a range of genetic diversity from a few genes to genome-scale screens. It integrates information from multiple phenotype measures to generate specific hypotheses for genetic interactions. We recently demonstrated the utility of this method in a population of yeast strains by mapping relationships of the yeast mating pathway (Carter *et al.* 2012). Here, we extend the method to a large set of double knockdowns of genes involved in signal transduction on a common genetic background (Horn *et al.* 2011). This represents a much larger network of potential interacting genes and involves multiple signaling pathways. The analysis exploits the subtle differences between the regulation of cell proliferation and nuclear size across 93 mutations. We obtain an interaction network of knockdown-to-knockdown influences that identifies novel suppressors and enhancers of Ras signaling, demonstrating the method’s applicability to complex networks and large-scale genetic screens of multiple phenotypes. At a local level, the network provides specific hypotheses of how each gene knockdown modifies the activity of other gene knockdowns in the same pathway and across antagonistic pathways, narrowing the possible molecular mechanisms that underlie the observed genetic interactions.

## Methods

### Data source

Data were obtained from a study of double-stranded RNA (dsRNA) gene knockdowns (Horn *et al.* 2011) that is briefly summarized as follows. Schneider S2 cells were exposed to two independent dsRNA molecules to knock down 93 genes in all 4278 pair-wise combinations. Genes were chosen based on prior annotation for mitogen-activated protein kinase (MAPK) signaling or prior annotation as a protein or lipid phosphatase expressed in S2 cells. High-throughput fluorescent imaging and automated image analysis were used to quantify three non-redundant features: total cell number, mean nuclear area per cell, and nuclear fluorescence intensity. Data for each knockdown pair were averaged and log-transformed, and each phenotype distribution was mean-centered and normalized to a standard deviation of 1. At a population level, each phenotype resembled the combination of a normal distribution and a long tail below the mean that was populated by lines with knockdowns of a few especially strong perturbations such as *Pvr*, *pnt*, and *drk* (Table 1 and see Supporting Information, Figure S1).

**Table 1:** Genes with significant main effects used as covariates for all pair-wise scans, with prior pathway annotation (FlyBase 2004)

Knockdown	β^ET1^	β^ET2^	Pathway
*alph*		0.0093	Ras
*CG3573*		0.0141	Ras
*Cka*	−0.0126		Ras
*dome*	−0.0205		Ras
*drk*	−0.0373	0.0092	Ras
*Dsor1*	−0.0084	0.0209	Ras
*Gap1*	0.0152	−0.0099	Ras inhibitor
*lic*		−0.0105	JNK
*mop*	−0.0089		Ras
*msk*		−0.0095	
*mtm*	0.0086		Ras inhibitor
*mts*	−0.0255	0.0215	Ras
*pnt*	−0.0382	−0.0108	Ras
*PpV*	−0.0101		Ras
*Pten*	0.0110		
*Ptp69D*	0.0109		Ras inhibitor
*PTP-ER*	0.0087		Ras inhibitor
*puc*	−0.0153	0.0133	Ras
*Pvr*	−0.0600	−0.0189	Ras
*pyd*	0.0091		JNK
*Ras85D*	−0.0101	0.0108	Ras
*Rho1*	0.0139	0.0741	
*rl*	−0.0106	0.0106	Ras
*Sos*	−0.0104	0.0108	Ras
*Src42A*		−0.0163	JNK
*stg*	−0.0123	0.0220	Ras

β^ET1^, main effect on eigentrait 1; β^ET2^, main effect on eigentrait 2.

### Interaction model

Our data analysis technique is described at length in a previous publication (Carter *et al.* 2012) and is summarized here. We first perform singular value decomposition (SVD) on the phenotype matrix to maximize orthogonality. In this case, we analyzed the first two left singular vectors, referred to hereafter as eigentraits, since the third singular value represented less than 2% of the global variance and was therefore unlikely to encode significant biology (*Results*). With these eigentraits denoted $U_{i}^{1}$ and $U_{i}^{2}$ for sample *i*, we performed linear regression for each of the 93 perturbed genes in isolation to identify strong-effect knockdowns to be treated as additive covariates in subsequent pair-wise regressions. For each locus we considered the model:

$$U_{i}^{j}=\beta_{0}^{j}+x_{i}\beta^{j}+\varepsilon_{i}^{j}$$ (1)

The index *i* is from 1 to $N_{S},$ and j is 1 or 2. The variable $x_{i}$ is the probability of the gene variant at the locus in the strain *i*, $\beta^{j}$ is the effect on the eigentrait *i*, and $\varepsilon_{i}^{j}$ is the residual error. For our data all $x_{i}$ were binary, corresponding to the presence (1) or absence (0) of dsRNA that target the locus. We define strong-effect knockdowns as those with a significant effect (see below) and condition each subsequent knockdown-pair scan by including strong-effect knockdowns as covariates for the associated phenotype. We next model every possible knockdown pair with main effects and an interaction term. For two knockdowns labeled 1 and 2 we have:
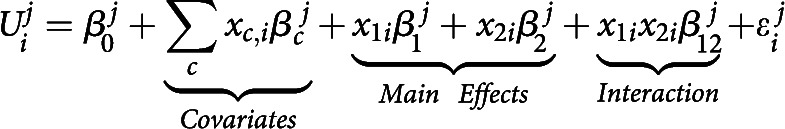
(2)The variables are defined as in Equation 1 with the additional interaction coefficient $\beta_{12}^{j}$ and sum over strong-effect knockdowns as covariates (excluding knockdowns 1 and 2). This step is conceptually similar to the original analysis of the data (Horn *et al.* 2011), which identified pair-wise interactions for each phenotype independently.

To derive a model in terms of knockdown-to-knockdown influences we consider how each knockdown affects the activity of each other knockdown, first in terms of modified knockdown activity and then in terms of the knockdown-to-knockdown influences that account for the modified activity level. We first recast the $N_{P}$ interaction coefficients $\beta_{12}^{j}$ in terms of modified knockdown activity parameters $\delta_{1}$ and $\delta_{2}$ that are independent of the eigentrait *j*. The activity variables are computed by matrix inversion:

$$\left[\begin{array}{c} \delta_{1}\\ \delta_{2} \end{array}\right]={\left[\begin{array}{cc} \beta_{1}^{1} & \beta_{2}^{1}\\ \beta_{1}^{2} & \beta_{2}^{2} \end{array}\right]}^{-1}\cdot \left[\begin{array}{c} \beta_{12}^{1}\\ \beta_{12}^{2} \end{array}\right]$$ (3)

The variables $\delta_{1}$ and $\delta_{2}$ are an exact reparametrization of the interaction coefficients. We next compute the directional influence coefficients that generate the modified activities. These knockdown-to-knockdown influences,$m_{12}$ and $m_{21}$, are:

(4) $$m_{12}=\frac{\delta_{1}}{1+\delta_{2}},m_{21}=\frac{\delta_{2}}{1+\delta_{1}}$$

By substituting the solution of Equation 3 into Equation 4 we obtain the influence coefficients $m_{ij}$ as a function of the regression parameters. This defines a model in terms of knockdown-to-knockdown influence coefficients ($m_{ij}$) and main-effect, knockdown-to-eigentrait coefficients ($\beta_{i}^{j}$). Without loss of information, we multiply the eigentrait coefficient matrix by the other center and left singular value matrices to recompose knockdown-to-phenotype coefficients for the original phenotypes.

### Calculation of significance

We assess the significance of the influence coefficients, $m_{ij}$, and knockdown-to-phenotype coefficients using standard error analysis methods on the regression parameters (Bevington 1969). For example, the variance of $m_{12}$ is estimated by differentiating with respect to all model parameters:

$$\sigma_{m_{12}}^{2}≅\underset{i,j}{\sum }\sigma_{\beta_{i}^{j}}^{2}{\left(\frac{\partial m_{12}}{\partial \beta_{i}^{j}}\right)}^{2}+2\underset{i<k,\;j<l}{\sum }\sigma_{\beta_{i}^{j}\beta_{k}^{l}}^{2}\left(\frac{\partial m_{12}}{\partial \beta_{i}^{j}}\right)\left(\frac{\partial m_{12}}{\partial \beta_{k}^{l}}\right)$$ (5)

The indices i and k run over main effect and interaction coefficients, and j and l run from 1 to $N_{P}$. The first sum is over individual parameters and the second double sum is the cross terms. Variances and covariances are estimated from the least-squares regression using standard methods (Bevington 1969).

After computing models for all knockdown pairs, we obtained two knockdown-to-knockdown influence coefficients between each knockdown pair (one in each direction) and knockdown-to-phenotype coefficients. To determine significance we used effect size divided by estimated standard error as our test statistic because it could be computed for both regression coefficients and variables computed from them (*e.g.*, $m_{ij}$). We selected the coefficient of median standard effect size to represent overall knockdown-to-phenotype coefficients. We first determined significance for single-locus scans. We performed 2000 permutations of the genotype data and fit an extreme value distribution (EVD) for the maximum test statistic from each permutation. This accounted for multiple tests and empirically estimated the likelihood of chance association. We determined that a test statistic of 5.18 or greater corresponds to *P* < 0.001. We repeated the procedure for pair-wise scans, permuting the genotypes of the two knockdowns being tested in tandem. This procedure retained the structure between all other knockdowns (including covariates) and all three phenotypes, and thereby randomized only the marginal effects after conditioning on covariates. We collected test statistics for 700 permutations to obtain null distributions for knockdown-to-phenotype and knockdown-to-knockdown influence coefficients. We computed an empirical *P* value for each coefficient. The family wise error rate was controlled by adjusting *P* values using a step-down procedure (Holm 1979). Step-down EVDs were not used because the empirical distributions had slightly greater support at higher values than fitted EVDs and thus the EVDs would artificially inflate significance. A significance cutoff of adjusted *P* < 0.01 was used in our network (Figure 3) and all estimated *P* values are reported in Table S1.

## Results

**Figure 1:**
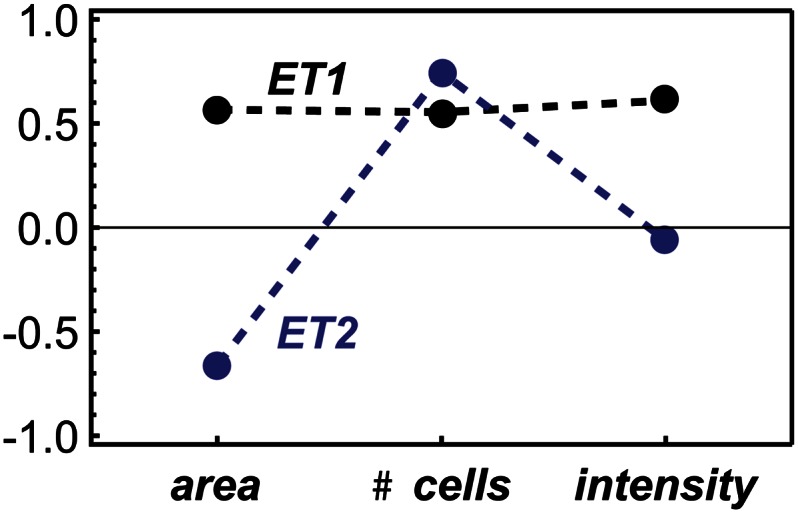
Eigentrait compositions in terms of measured phenotypes. Eigentrait 1 (ET1) is the signal common to all three phenotypes, whereas eigentrait 2 (ET2) encodes the differences.

The assayed phenotypes shared many genetic components and therefore exhibited substantial pleiotropy. All three phenotypes were significantly correlated, with Pearson coefficients as follows: cell number and nuclear area, 0.65; cell number and nuclear intensity, 0.90; and nuclear area and nuclear intensity, 0.85. However, there was variation across the tested knockdowns. For example, the *Rho1* defect in cytokinesis caused decreased cell numbers and increased nuclear area, whereas Ras signaling knockdowns such as *drk* decreased both numbers and area (Horn *et al.* 2011). To maximize complementarity and dimensionally reduce the phenotype data, we performed singular value decomposition (SVD) to define two composite eigentraits, each an orthogonal combination of the phenotypes (Methods). The eigentraits were orthogonal, normalized combinations of the phenotypes (Figure 1) and represented 87%, 11.5%, and 1.5% of the variance in the data. Since the bulk of the variance was present in the first two eigentraits we discarded the third. We refer to these composite phenotypes as eigentrait 1 (ET1) and eigentrait 2 (ET2). ET1 is a common signal to all three phenotypes, whereas ET2 primarily differentiates nuclear area from cell number and nuclear intensity. Thus, the inferences made for genetic interactions will be mostly based on combining an overall common signal with a signal that separates nuclear area from the other two, highly correlated phenotypes.

For each eigentrait we performed linear regression for each knockdown individually, similar to the method in the original study (Horn *et al.* 2011). This identified significant knockdowns to be used as covariates in pair-wise scans (Methods). We identified 21 knockdowns as covariates for ET1 and 17 knockdowns for ET2 (Table 1, Figure S1 and Figure S2). We next computed a pair-wise model for each of the 4278 knockdown pairs to obtain quantitative interactions between knockdowns (Table S1). We reparametrized the two interaction coefficients from pair-wise linear regression on the eigentraits (Methods, Equation 2) in terms of two knockdown-to-knockdown influences between perturbations (Methods, Equation 4) (Carter *et al.* 2012). To determine the allelic effects on the original cellular phenotypes, we recomposed the phenotype SVD for each pair-wise model and averaged all models to obtain knockdown-to-phenotype influence coefficients (*Methods*). Errors were estimated for all coefficients using standard least-squares regression and error propagation formulas for quantities computed from regression coefficients (Methods, Equation 5).

**Figure 2:**
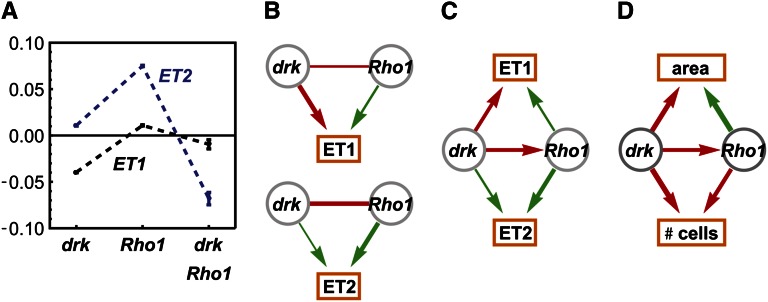
Genetic interactions between *drk* and *Rho1* knockdowns. (A) Inferred main and interaction effects of *drk* and *Rho1* mutations on eigentraits ET1 and ET2. (B) Significant main and interaction effects shown in terms of positive (green) and negative (red) influences. Edge width represents interaction strength. (C) Interaction model consistent with both ET1 and ET2, in which *drk* knockdown suppresses *Rho1* knockdown. (D) The same model expressed in terms of the original phenotypes of total cell number and nuclear area (total fluorescent intensity is similar to cell number and not shown).

**Figure 3:**
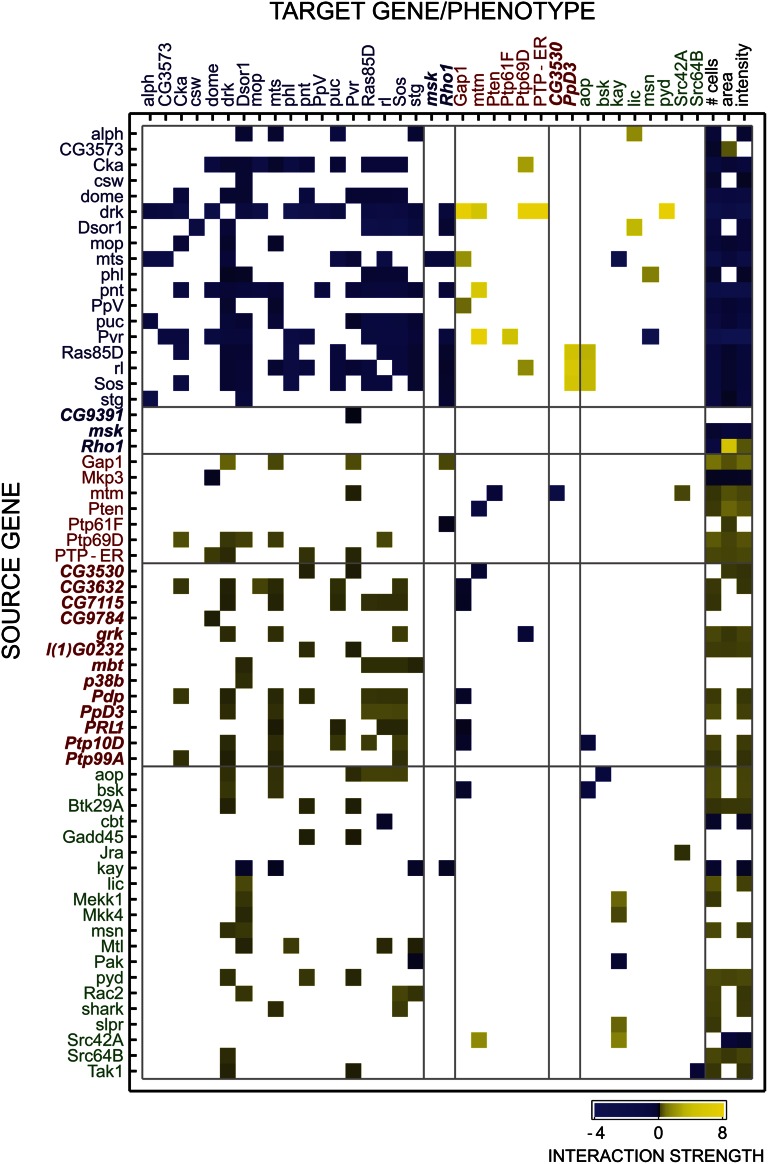
Adjacency matrix of significant interactions for knockdowns of Ras (blue labels), Ras inhibitors (red labels), and JNK (green labels) genes. Previously uncharacterized gene names are in italics. Color denotes interaction sign and intensity of directional interactions. Overall, within-pathway interactions are overwhelmingly suppressive (blue squares), whereas interactions across antagonistic pathways are enhancing (yellow squares). Ras knockdowns generally reduce cellular phenotypes, while Ras inhibitors and JNK knockdowns increase phenotypes. Empty squares did not meet the significance threshold of *P* < 0.01 (see text).

A representative interaction is illustrated in Figure 2. The *drk* and *Rho1* knockdowns have opposite effects on the first eigentrait (Figure 2A) and a negative regression interaction coefficient (Figure 2B). For the second eigentrait, both knockdowns have positive effects and the regression interaction coefficient is negative and larger than that for ET1. Reparametrizing these results in terms of our genetic influence coefficients inferred a significant negative influence from *drk* to *Rho1* (Figure 2C); that is, the *drk* knockdown suppressed the effects of the *Rho1* knockdown. The complementary information in the two eigentraits ruled out alternative hypotheses such as a *Rho1* enhancement of the *drk* effect that would have been consistent with ET1 but not ET2. Finally, we recomposed the eigentraits into the original phenotypes (Figure 2D), an operation that does not modify the inferred genetic interaction. This analysis recapitulates the interpretation in Horn *et al.* (2011) with the advantage of a mathematically defined procedure that has been systematically applied to all pairs of knockdowns in the data set. In total, we obtained a network of 290 significant knockdown-to-knockdown edges and 146 significant knockdown-to-phenotype edges. Interactions for 61 genes with existing or novel (see below) functional annotations are shown in Figure 3.

Sixty-three knockdowns had a significant main effect (*P* < 0.01; Methods) on at least one of the three phenotypes, and 29 had significant influences on all three phenotypes (Figure 3). Knockdowns of the 18 genes previously annotated for Ras signaling had 48 negative effects, compared to only one positive effect [*CG3573* (*Ocrl*) knockdown on nuclear area]. In contrast, knockdowns of the 29 genes previously annotated for JNK cascade and Ras inhibition had 48 positive main effects, compared to 13 negative effects. These findings are consistent with Ras signaling activation of cell proliferation and growth and the generally antagonistic relationship between Ras and JNK signaling. For each knockdown, main effects were generally consistent across all partners in each pair-wise scan; no specific combination of knockdowns revealed a uniquely significant effect for one of the genes.

Our total of 290 significant knockdown-to-knockdown interactions out of 8556 possible (two for each pair) was numerically comparable to the 531 significant interactions out of 12,834 possible (three phenotypes for each pair) obtained with the same threshold in the previous study (Horn *et al.* 2011). Gene pairs with at least one significant interaction in both analyses strongly overlapped, as 55% (126 of 229 pairs) of our significant pairs were also significant in at least one phenotype in Horn, *et al.* (8 × 10^−88^, Fisher’s exact test). Pairs that did not overlap nevertheless tended to have low *q* values in Horn, *et al.* For each of our pairs we identified the lowest *q* value across all three phenotypes, and found a median of 0.006 as compared to a global median lowest *q* value of 0.38. This demonstrates that our analysis generally agreed with more standard measures of epistasis and, in some cases, improved significance by reformulation in terms of directional model parameters.

Global genetic interaction patterns revealed large-scale gene function and module/pathway organization (Figure 3 and Figure S3). Knockdown-to-knockdown influences between knockdowns of documented Ras signaling genes were fairly common (63% of possible interactions were significant) and always negative. We interpret this as genes with redundant signaling functions. Although it is suggestive that downstream knockdowns should suppress upstream knockdowns (Avery and Wasserman 1992; Carter *et al.* 2012), the majority of pair-wise knockdowns of known Ras genes exhibited significant negative interactions in both directions. In cases of unidirectional suppression, some known downstream knockdowns (*e.g.*, *pnt*) tended to suppress upstream proteins (*e.g.*, *Ras85D* and *Sos*) but others (*e.g.*, *rl*) were suppression targets more often than not. Overall, there is a clear pattern of negative interactions between within-pathway genes. In contrast, Ras inhibitor knockdowns tended to enhance the effect of knockdowns of Ras signaling genes. This is consistent with standard interpretations of genetic interactions in signaling. For example, *PTP-ER* is named for its mutant alleles that enhance Ras signaling in eye development (Karim and Rubin 1999). Furthermore, knockdowns of genes documented or predicted to be involved in JNK signaling generally enhanced Ras mutations, consistent with the antagonistic relationship between JNK and Ras signaling.

**Figure 4:**
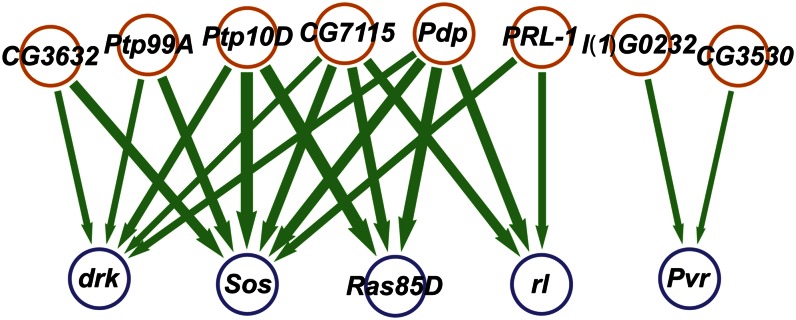
Network of novel candidate Ras inhibitor phosphatases and Ras kinases inferred from interaction patterns. Knockdowns of phosphatase genes (orange nodes) enhance the effects of Ras kinase knockdowns (blue nodes), similar to known Ras inhibitors and JNK signaling genes (Figure 3). Edge width denotes relative strength of enhancement.

Specific edges in the interaction network allowed us to infer the function of many uncharacterized genes, based on the rule that knockdowns functioning in the same signaling process will exhibit suppressive interactions whereas knockdowns functioning in antagonistic processes will exhibit enhancing interactions (Avery and Wasserman 1992). We assessed the interactions for 45 genes not previously associated with Ras or JNK signaling and one gene (*Rho1*) which had been annotated for both (Flybase 2004; Noselli and Agnes 1999). Novel pathway assignments were made when an unannotated knockdown exhibited interactions with Ras knockdowns annotated in Horn, *et al.* (Horn *et al.* 2011) or curated data (Flybase 2004). Negative interaction partners were candidate Ras signaling genes while positive interaction partners were designated as Ras inhibitors. We assigned Ras signaling function to three knockdowns that suppressed the effect of one or more Ras signaling knockdowns and/or had its effect enhanced by one or more Ras inhibitor knockdowns (Table 2). These novel Ras signaling knockdowns had generally negative influences on cell number and nuclear area (Figure 3), consistent with other Ras signaling knockdowns. The multiannotated gene *Rho1* was in this group, suggesting that for the measured phenotypes it operates as a Ras signaling component. We assigned Ras inhibitor function to 13 knockdowns that enhanced the effect of one or more Ras signaling knockdowns and/or had its effect enhanced by one or more Ras signaling knockdowns (Table 2 and Figure 3). In most cases these novel Ras inhibitor knockdowns suppressed the knockdown of a fellow Ras inhibitor knockdown and the vast majority had a positive influence on cell number and nuclear area (Figure 3), consistent with other Ras inhibitors. Ten of these genes are annotated as phosphatases and, in many cases, we identified specific Ras signaling kinases for which knockdown effects are unidirectionally enriched by one or more phosphatase knockdowns (Figure 4). These provide specific hypotheses for the prioritization of phosphatase-kinase targeting experiments. The inferred Ras-inhibitor phosphatase *PpD3* exhibited mutual enhancing interactions with Ras knockdowns *Sos*, *Ras85D*, and *rl* (Figure 3), which suggests a more complex role in Ras inhibition. Taken in sum, these 16 new functional hypotheses can be added to the set of approximately 20 novel pathway assignments inferred in the original, classifier-based analysis of the interaction data (Horn *et al.* 2011).

**Table 2:** New candidate Ras signaling genes and Ras inhibitors, with relevant GO annotations for the knockdown (Ashburner *et al.* 2000; FlyBase 2004)

Pathway	Knockdown	Interaction Partner(s)	Annotated Function
Ras signaling	*CG9391*	*Pvr*	Inositol monophosphate 1-phosphatase
	*msk*	*mts*	Ran GTPase binding, protein transport
	*Rho1*	*drk*, *Dsor1*, *mts*, *pnt*, *Pvr*, *Ras85D*, *rl*, *Sos*, *stg*	Kinase binding (JNK cascade and GTPase signal transduction both predicted)
Ras inhibitor	*CG3530*	*pnt*, *Pvr*	Protein tyrosine/serine/threonine phosphatase
	*CG3632*	*Cka*, *drk*, *mop*, *mts*, *puc*, *Sos*	Protein tyrosine/serine/threonine phosphatase
	*CG7115*	*drk*, *mts*, *puc*, *Ras85D*, *rl*, *Sos*	Protein serine/threonine phosphatase, cell adhesion
	*CG9784*	*dome*	Inositol trisphosphate phosphatase
	*grk*	*drk*, *mts*, *Sos*	Epidermal growth factor receptor binding
	*l(1)G0232*	*pnt*, *Pvr*	Non-membrane spanning protein tyrosine phosphatase
	*mbt*	*Dsor1*, *Ras85D*, *rl*, *Sos*, *stg*	Protein serine/threonine kinase, negative regulation of cell size
	*p38b*	*Dsor1*	Protein serine/threonine MAP kinase, stress response, positive regulation of cell size
	*Pdp*	*Cka*, *drk*, *mts*, *pnt*, *Ras85D*, *rl*, *Sos*	Protein serine/threonine phosphatase
	*PpD3*	*drk*, *mts*, *Ras85D*, *rl*, *Sos*	Protein serine/threonine phosphatase, mitotic cell cycle
	*PRL-1*	*mts*, *puc*, *rl*, *Sos*	Prenylated protein tyrosine phosphatase
	*Ptp10D*	*drk*, *mts*, *puc*, *Ras85D*, *Sos*	Protein tyrosine phosphatase, central nervous system development
	*Ptp99A*	*Cka*, *drk*, *mts*, *Sos*	Transmembrane receptor protein tyrosine phosphatase, motor axon guidance

GO, gene ontology.

## Discussion

We have used a strategy that combines patterns of epistasis and pleiotropy to derive a large-scale network mapping how mutations in signaling genes interact to affect distinct phenotypes. The approach integrates information in multiple phenotypes to resolve ambiguities in model interpretation that arise when individual phenotypes are analyzed in isolation. We have demonstrated the applicability of this strategy to large-scale data, in this case involving 4278 cell lines that mix 93 genetic perturbations. Significant knockdown-to-knockdown interactions were obtained among 68 knockdowns even though each was fairly rare in the population (present in 2.2% of lines). While the original analysis of these data (Horn *et al.* 2011) revealed patterns of genetic interactions separately for each phenotype, this analysis combines the multiphenotype patterns of epistasis to derive directional models of genetic interactions that are consistent across all three phenotypes. This additional mathematical analysis recovered previous findings and was used to propose additional functional relationships between gene pairs.

Many of the interactions in the network (Figure 3) recapitulated known relationships. Genes that function in the same pathways show consistent, suppressive genetic interactions across a spectrum of interaction partners. This is consistent with the idea that mutations with redundant effects suppress each other, particularly in signaling pathways. The spectral consistency for interactions, which has been previously referred to as “genetic monochromaticity” (Segre *et al.* 2005), is maintained after their reparametrization in terms of directional influences. A similar spectral consistency is demonstrated for enhancing interactions from pairs spanning multiple pathways. We interpret this enhancement as a signature of an antagonistic relationship. For instance, a knockdown of a Ras inhibitor will increase Ras signaling activity, and therefore a subsequent Ras knockdown will have an enhanced effect since the signaling baseline is increased (*cf*. *PTP-ER*, Results). Following this interpretation, in addition to spectral consistency, these interactions can map activation and suppression across the Ras and JNK pathways. The specificity represents *de novo* derivation of key functional relationships that are more difficult to detect when analyzing each phenotype independently.

We note that classical synthetic effects, in which two perturbations with insignificant main effects generate a strong effect in combination, would also produce enhancing interactions. We did not observe any such cases in our data. When we broaden the definition to include knockdowns with main effects and greater than additive combinatorial effects, all with the same sign (positive or negative), we find only two candidate interactions. *Gap1* knockdown enhances *Rho1* knockdown activity and both positively affect nuclear area and nuclear intensity; however they show opposite effects on cell number and therefore the effect is not consistently synthetic (Figure 3). A less ambiguous candidate is the strong enhancement of *kay* knockdown effects by *Src42A* knockdown. Both perturbations significantly decrease nuclear intensity, and this effect becomes significantly greater when the knockdowns are combined. These genes, both annotated as JNK pathway elements, provide the only unambiguous instance of synthesis in the data.

Our analysis provides new and refined information on gene-gene relationships and places this information in a network context. At a single-interaction level, we identified novel candidate Ras inhibitors and coactivators (Table 2 and Figure 4). At a network-wide level, these genes were spectrally consistent with other genes of similar known function (Figure 3 and Figure S3). Thus, although prior information is usually helpful in placing new candidate mutations in a functional context, the interactions we inferred represent a *de novo* model of enhancement or suppression with functional implications. Furthermore, some of the network inferences propose refinements of current knowledge, such as the hypothesis that *Rho1* is primarily a Ras signaling gene in the context of the measured phenotypes. Likewise, the interaction patterns and phenotype influences of the *kayak* (*kay*) knockdown suggest that its functional role in cell proliferation is more similar to Ras signaling genes than JNK cascade genes, in contrast to its similarity to JNK in dorsal closure (Harden 2002). Specifically, the *kay* knockdown resembled Ras signaling genes by suppressing the knockdown effect of multiple Ras signaling genes, being enhanced by the knockdown of multiple JNK cascade genes, and having a negative main effect on cell number (Figure 3).

In particular, our analysis suggested novel Ras inhibitors not detected the previous study using the same data (Horn *et al.* 2011). In most cases, inhibitor knockdowns redundantly enhanced multiple kinases (Figure 4). This could be due to non-specific phosphatase activity, but is potentially a result of functional redundancy among the kinases that leads to similar genetic interactions between a phosphatase and both its direct target and genes that cofunction with that target in a signaling cascade. One possible avenue to resolve this ambiguous redundancy would be to extend the interaction analysis beyond independent pair-wise analysis to models that find the most parsimonious model for three or more genes (Carter *et al.* 2007). We note that the majority of these phosphatase knockdowns significantly suppressed the *Gap1* knockdown, providing evidence of redundant function with a known *Ras85D* inhibitor. We also note that a number of additional enhancing interactions from phosphatases to kinases that did not meet our significance threshold were also detected, providing additional evidence of functional coherence but target non-specificity. However, there does seem to be limited specificity in that *CG3632* and *Ptp99A* enhance *drk* and *Sos*, genes that fall relatively upstream in the KEGG-annotated Ras pathway, while *CG7115* and *Pdp* knockdowns most strongly enhance the relatively downstream pathway knockdown *Ras85D*. In contrast, phosphatase knockdowns *l(1)G0232* and *CG3530* enhance the *Pvr* knockdown, which is not placed in the KEGG-annotated Ras cascade. The additional information that the *Pvr* knockdown tends to unidirectionally suppress the KEGG pathway genes suggests that *Pvr* potentially acts downstream of the KEGG-annotated pathway and is deactivated by the phosphatases *l(1)G0232* and *CG3530*. Thus, even with the ambiguities arising from pair-wise models, we obtained a degree of specificity in hypotheses for Ras inhibitor activity.

The network of directional influences suggests hypotheses that are often amenable to experimental testing (Carter *et al.* 2007). In contrast with non-directional associations such as coexpression, statistical epistasis, or synthetic lethality, a directional influence identifies a driver mutation that affects the activity of its target, a second gene that, in turn, affects the phenotype. With this in mind, we can predict that removing the influence with an experimental perturbation, such as an exogenous inhibitor molecule, would nullify the effect of mutations in the target gene. Furthermore, the effect type (enhancement or suppression) specifies the nature of the effect on the target gene. We can therefore predict that overexpression of a novel Ras inhibitor will not only affect, but specifically reduce the relevant activity of its enhanced targets, whereas perturbations of the targets are not expected to affect the Ras inhibitor. For example, overexpression of *Ptp99A* will specifically inhibit the activity of Ras signaling proteins *Cka*, *drk*, *mts*, and *Sos*. In this way, a network of directional genetic interactions could prove useful in the selection of candidate genes for validation and, in disease-related networks, the prioritization of therapeutic targets.

Our analysis was limited by the genotypes and specific phenotypes selected in the original study. Although the three phenotypes used exhibit differential interaction patterns (Horn *et al.* 2011), their high correlation limited the complementary information that could better resolve directional interactions. By probing additional phenotypes, new interactions might be detected and existing interactions refined. For instance, we speculate that mutual cosuppression among Ras signal knockdowns is potentially due to measuring insufficiently distinct outputs. Combining detailed assays of eye development and terminal patterns might have revealed more specific roles and pathway order for canonical Ras signaling proteins. Furthermore, although the genetic perturbations are limited to MAPK signaling genes, the methods could be applied to a wider screen or population-based analysis to identify additional pathway relationships. As data collection in experimental systems and clinical studies becomes more diverse and quantitative, our analytical method has the potential to derive more refined and precise network models.

The approach we have applied derives quantitative, directional models of how each gene knockdown modifies the effects of each other knockdown. It represents an alternative to guilt-by-association methods that infer function by scoring correlations across large-scale data. Taken together as a network, the interactions form consistent patterns between and within gene modules. Large-scale data and prior information is helpful but not necessary because guilt precedes association in our analysis. This allowed us, for instance, to propose new candidate Ras inhibitors that are consistent with known Ras inhibitors, but do not necessarily depend on this prior knowledge for functional inference.

## Supplementary Material

Supporting Information
