# Supplementary material for: Inferring Gene Function and Network Organization in Drosophila Signaling by Combined Analysis of Pleiotropy and Epistasis
Source: G3 (Bethesda). 2013 May 1;3(5):807–14. doi: 10.1534/g3.113.005710 (PMC3656728; doi:10.1534/g3.113.005710)
Supplement: Supporting Information [file supp_3_5_807__index.html]

Inferring Gene Function and Network Organization in Drosophila Signaling by Combined Analysis of Pleiotropy and Epistasis — Supporting Information 

# Inferring Gene Function and Network Organization in *Drosophila* Signaling by Combined Analysis of Pleiotropy and Epistasis

## Supporting Information for Carter, 2013

**Files in this Data Supplement:**

- Supporting Information - Figures S1-S4 and Table S1 (PDF, 305 KB)
- Figure S1 - Main effect coefficients (βiET1) of each gene knockdown for single-locus scans of eigentrait 1 (ET1) (PDF, 99 KB)
- Figure S2 - Main effect coefficients (βiET2) of each gene knockdown for single-locus scans of eigentrait 2 (ET2) (PDF, 99 KB)
- Figure S3 - Median influences from knockdowns in one functional group (rows) to knockdowns in other groups and phenotypes (columns) (PDF, 133 KB)
- Figure S4 - Adjacency matrices of direct effects (left columns) and significance (right columns; effect size divided by standard error) comparing non-interacting (1d) and interacting (2d) models (PDF, 113 KB)
- Table S1 - Results for all computed influence parameters. (.xlsx, 4.3 MB)
