# Supplementary material for: Inferring Gene Function and Network Organization in Drosophila Signaling by Combined Analysis of Pleiotropy and Epistasis
Source: G3 (Bethesda). 2013 May 1;3(5):807–14. doi: 10.1534/g3.113.005710 (PMC3656728; doi:10.1534/g3.113.005710)
Supplement: Supporting Information [file supp_g3.113.005710_005710SI.pdf]

G3 · Genes | Genomes | Genetics

Inferring Gene Function and Network Organization in Drosophila Signaling by Combined Analysis of Pleiotropy and Epistasis

Gregory W. Carter

The Jackson Laboratory
600 Main Street
Bar Harbor, ME 04609
Telephone: 207-288-6025
Email: greg.carter@jax.org

DOI: 10.1534/g3.113.005710

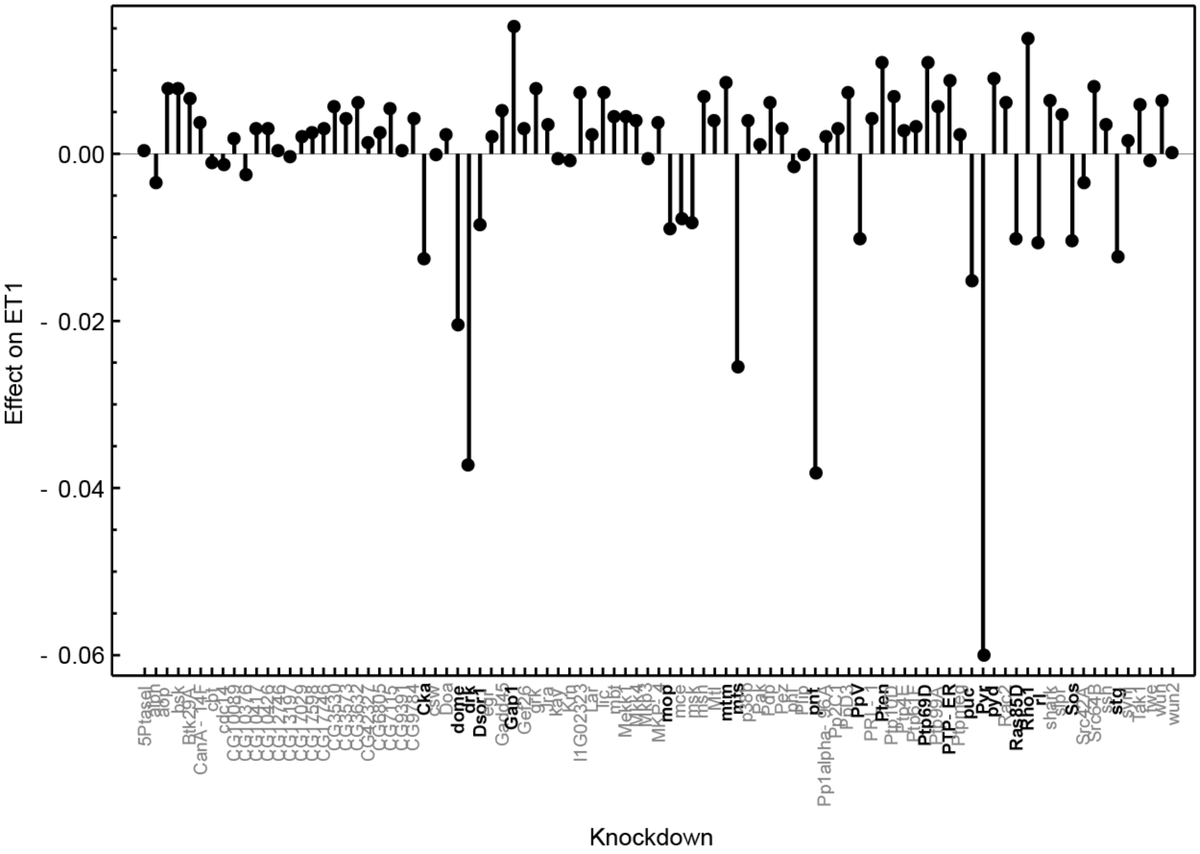

Figure S1 Main effect coefficients (β_i^{ET1}) of each gene knockdown for single-locus scans of eigentrait 1 (ET1). Knockdowns with significant effects are labeled in bold.

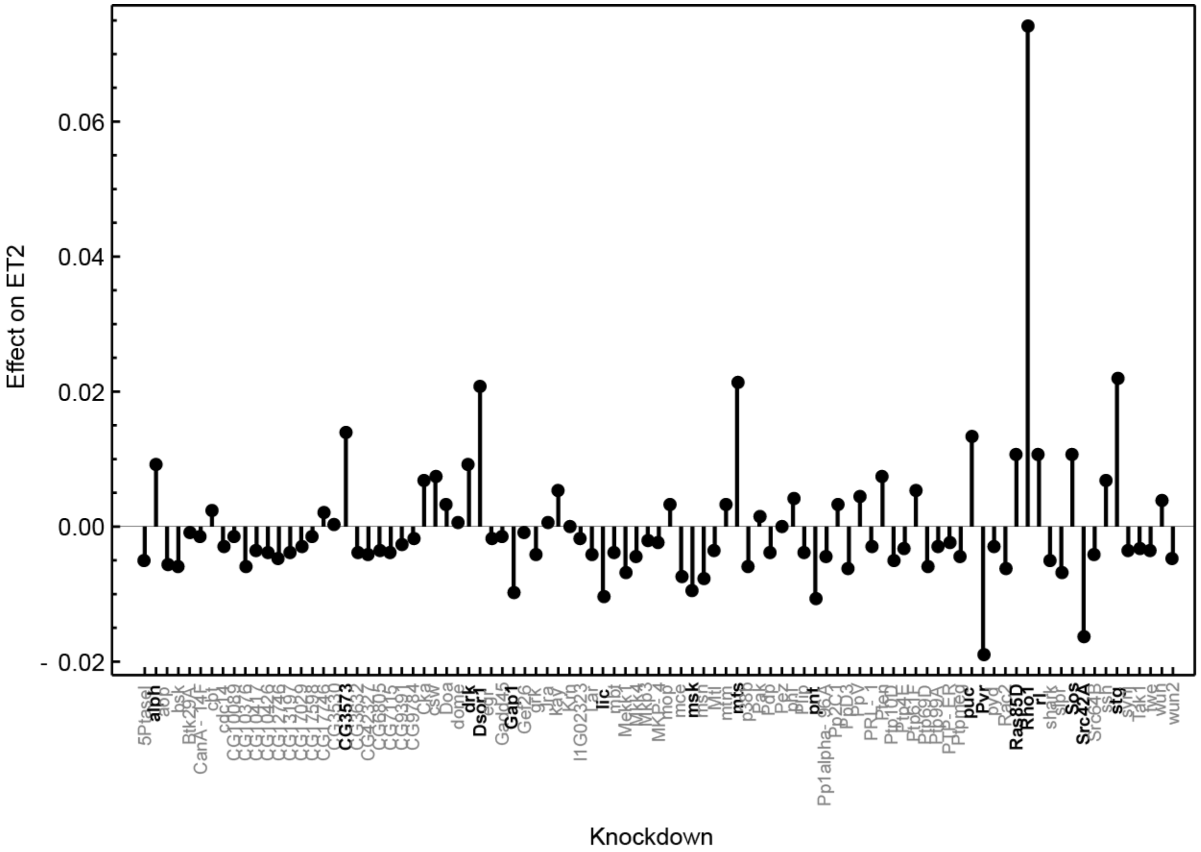

Figure S2 Main effect coefficients (β_i^{ET2}) of each gene knockdown for single-locus scans of eigentrait 2 (ET2). Knockdowns with significant effects are labeled in bold.

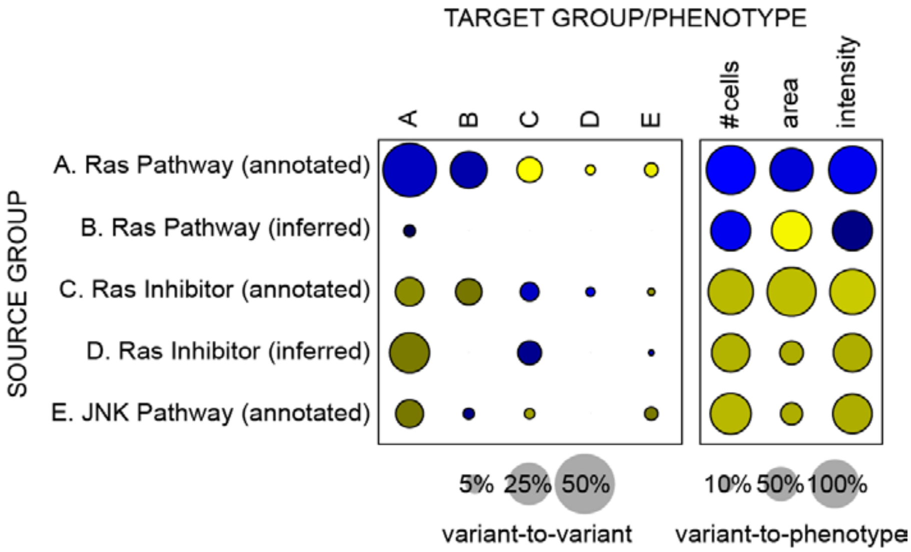

Figure S3 Median influences from knockdowns in one functional group (rows) to knockdowns in other groups and phenotypes (columns). Disc shading represents positive (yellow) or negative (blue) relative intensity and radius denotes density of observed interactions as a percentage of possible interactions. Ras pathway knockdowns are consistently suppressed by other Ras knockdowns but enhanced by Ras inhibitors.

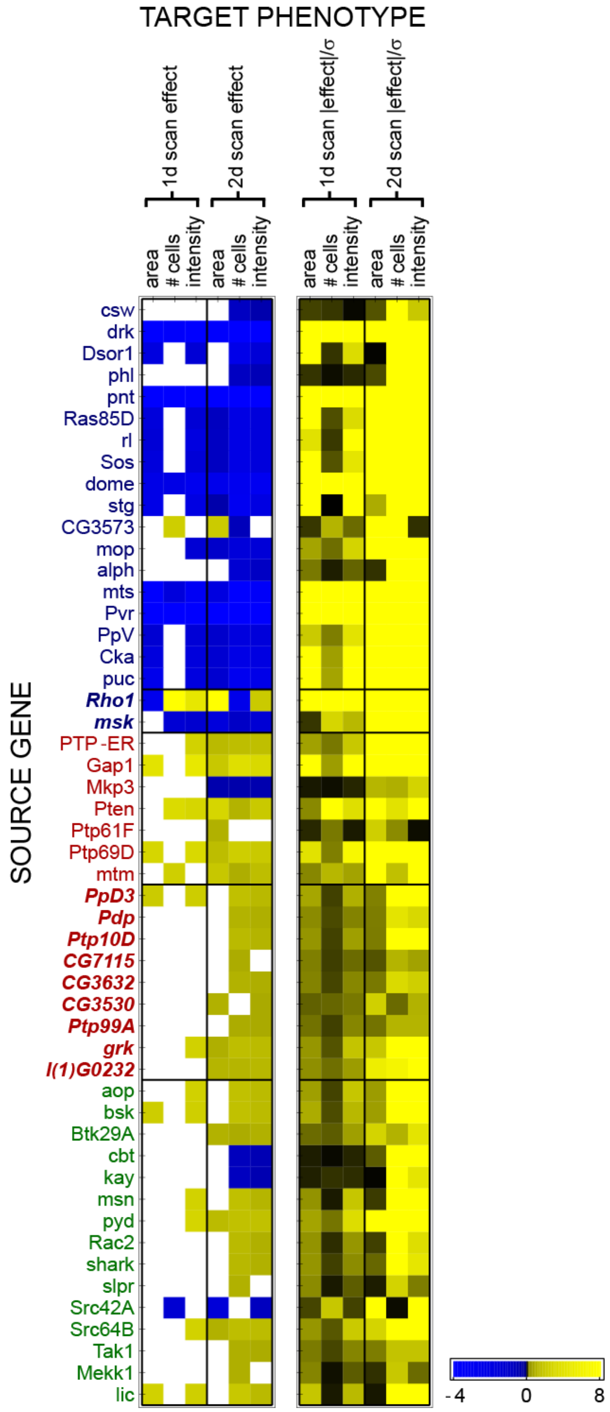

Figure S4 Adjacency matrices of direct effects (left columns) and significance (right columns; effect size divided by standard error) comparing non-interacting (1d) and interacting (2d) models. Genes are labeled as in Figure 3 and only significant interactions appear in effects (left columns). Interaction scans reveal additional direct influences of gene knockdowns on all three phenotypes by increasing significance of the inferred effects.

Table S1 Results for all computed influence parameters.

Table S1 is available for download at http://www.g3journal.org/lookup/suppl/doi:10.1534/g3.113.005710/-/DC1
