# Supplementary material for: Inferring Gene Function and Network Organization in Drosophila Signaling by Combined Analysis of Pleiotropy and Epistasis
Source: G3 (Bethesda). 2013 May 1;3(5):807–14. doi: 10.1534/g3.113.005710 (PMC3656728; doi:10.1534/g3.113.005710)
Supplement: Supporting Information [file supp_g3.113.005710_FigureS3.pdf]

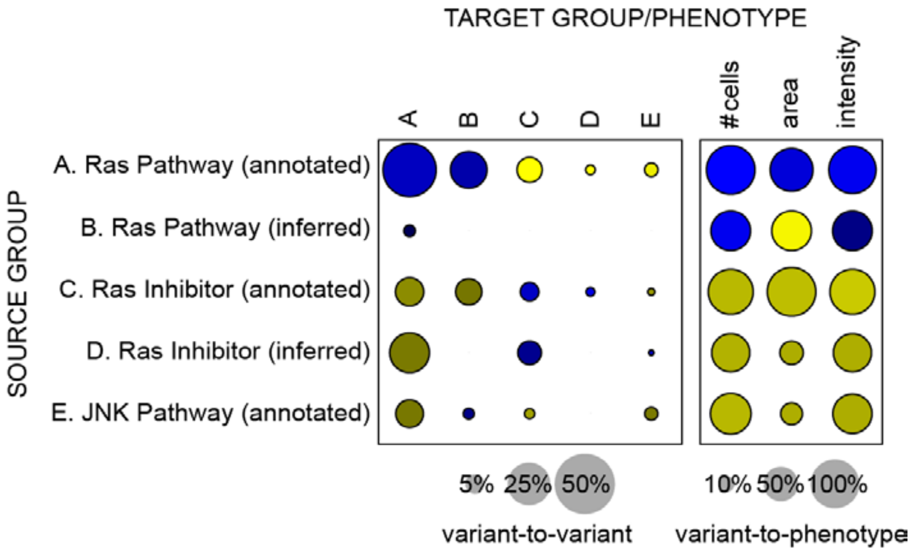

Figure S3 Median influences from knockdowns in one functional group (rows) to knockdowns in other groups and phenotypes (columns). Disc shading represents positive (yellow) or negative (blue) relative intensity and radius denotes density of observed interactions as a percentage of possible interactions. Ras pathway knockdowns are consistently suppressed by other Ras knockdowns but enhanced by Ras inhibitors.
