# Supplementary material for: Inferring Gene Function and Network Organization in Drosophila Signaling by Combined Analysis of Pleiotropy and Epistasis
Source: G3 (Bethesda). 2013 May 1;3(5):807–14. doi: 10.1534/g3.113.005710 (PMC3656728; doi:10.1534/g3.113.005710)
Supplement: Supporting Information [file supp_g3.113.005710_FigureS4.pdf]

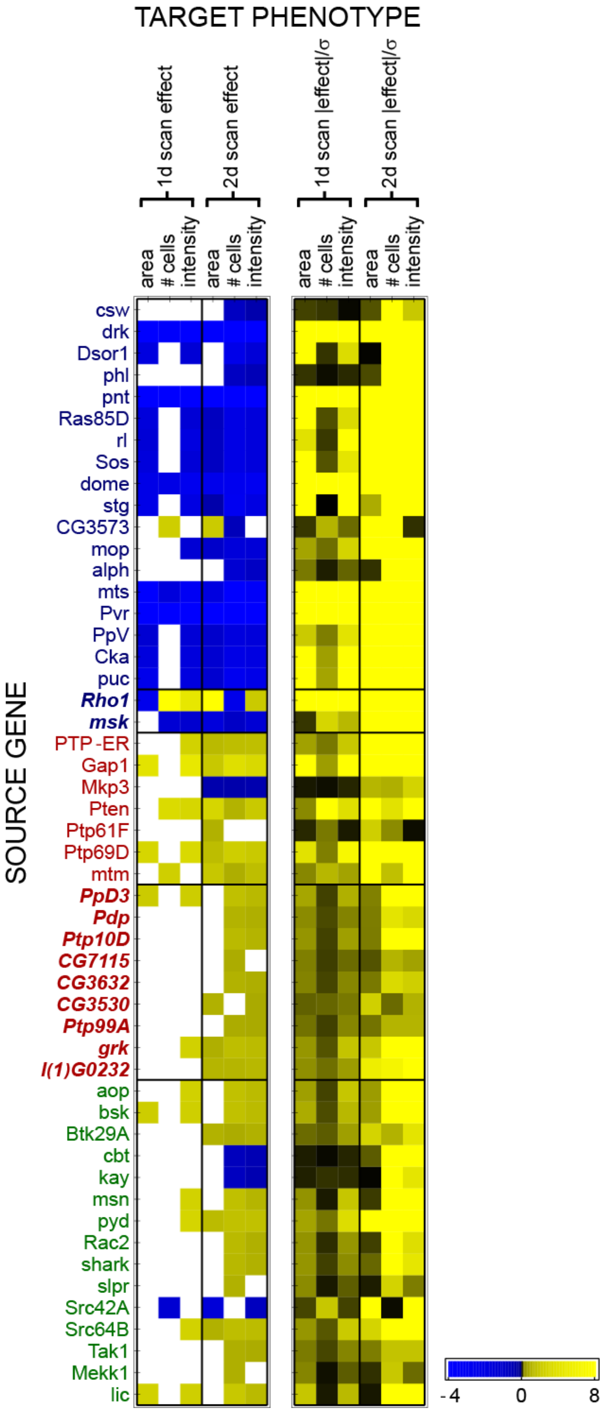

Figure S4 Adjacency matrices of direct effects (left columns) and significance (right columns; effect size divided by standard error) comparing non-interacting (1d) and interacting (2d) models. Genes are labeled as in Figure 3 and only significant interactions appear in effects (left columns). Interaction scans reveal additional direct influences of gene knockdowns on all three phenotypes by increasing significance of the inferred effects.
