# Supplementary material for: Inferring Gene Function and Network Organization in Drosophila Signaling by Combined Analysis of Pleiotropy and Epistasis
Source: G3 (Bethesda). 2013 May 1;3(5):807–14. doi: 10.1534/g3.113.005710 (PMC3656728; doi:10.1534/g3.113.005710)
Supplement: Supporting Information [file supp_g3.113.005710_FigureS1.pdf]

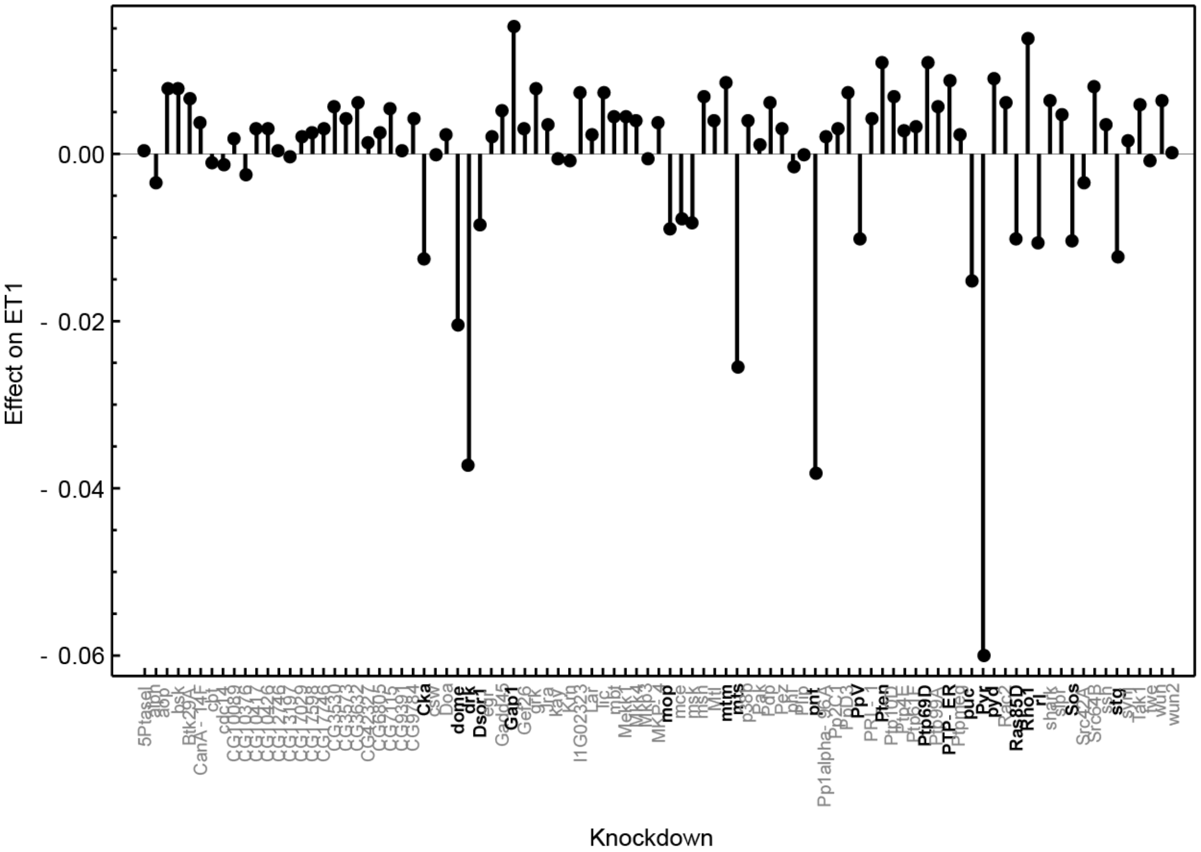

Figure S1 Main effect coefficients (β_i^{ET1}) of each gene knockdown for single-locus scans of eigentrait 1 (ET1). Knockdowns with significant effects are labeled in bold.
